# Molecular Design of Benzothiadiazole-Fused Tetrathiafulvalene Derivatives for OFET Gas Sensors: A Computational Study

**DOI:** 10.3390/s25196190

**Published:** 2025-10-06

**Authors:** Xiuru Xu, Changfa Huang

**Affiliations:** Key Laboratory of Optoelectronic Devices and Systems of Ministry of Education and Guangdong Province, College of Physics and Optoelectronic Engineering, Shenzhen University, Shenzhen 518000, China

**Keywords:** organic semiconductors, TTF derivatives, molecular design, frontier molecular orbitals, reorganization energy

## Abstract

**Highlights:**

- Theoretical calculations (PBE0/6-311G(d,p)) predict the designed promising organic semiconductors with high mobilities.
- TTF derivatives exhibit sensitivity to gases (NH_3_, H_2_S, SO_2_), suggesting potential for dual-function organic field-effect transistors (OFETs) with integrated sensing capabilities.

**Abstract:**

Due to their unique advantages—such as small size, easy integration, flexible wearability, low power consumption, high sensitivity, and material designability—organic field-effect transistor (OFET) gas sensors have significant application potential in fields such as environmental detection, smart healthcare, robotics, and artificial intelligence. Benzothiadiazole fused tetrathiafulvalenes (TTF) are promising organic semiconductor candidates due to their abundant S atoms and planar π-π conjugation skeletons. We designed a series of derivatives by side-chain modification, and conducted systematic computations on TTF derivatives, including reported and newly designed materials, to analyze how geometric factors affect the charge transport properties of materials at the PBE0/6-311G(d,p) level. The frontier molecular orbitals (FMOs) and reorganization energy indicate that the designed derivatives are promising candidates for organic semiconductor sensing materials. Furthermore, theoretical calculations reveal that the designed TTF derivatives are sensitive to gases like NH_3_, H_2_S, and SO_2_, indicating organic field-effect transistors (OFETs) with gas-sensing functions.

## 1. Introduction

Organic field-effect transistor (OFET) gas sensors hold groundbreaking potential across various advanced fields due to their flexibility, wearability, low power consumption, high sensitivity, and customizable materials. They are valuable not only in traditional areas like environmental monitoring but also play a vital role in robotics, artificial neural networks, electronic nose technology, and more. OFET gas sensors are expected to surpass the limitations of conventional gas detection methods and enable smarter, more interactive perception systems [1,2]. Developing high-performance gas-sensitive organic semiconductors (OSCs) is essential to improve OFET device performance [3]. To date, many organic semiconductor materials have been reported, including rubrene, anthracene, tetrobenzene, and pentacene [4]. Among these, TTF and its derivatives have gained widespread interest over the past decades because of their excellent charge transport in OFETs, highly tunable properties, and gas-sensing properties [5,6,7]. The mobility of TTF derivatives can reach as high as 11 cm^2^V^−1^s^−1^ (HM-TTF, Hexamethylenetetrathiafulvalene) [8]. Single crystals of DT-TTF show high mobility up to 3.6 cm^2^V^−1^s^−1^ [9]. TTF and its derivatives are promising candidates for high-quality organic semiconductors because of three key features: (1) an almost coplanar conjugated structure and abundant S atoms that promote π-π stacking and strong intermolecular interactions [10]; (2) ease of dissolving in organic solvents, enabling low-cost, large-scale production through solution processing [11,12]; and (3) the ability to modify the molecular structure through chemical synthesis [13]. However, due to TTF’s strong electron-donating properties, it easily oxidizes and can be doped by oxygen and water in the air, which may impact device stability [14]. To address this, side chain modifications, such as the addition of stabilizing groups, can help improve both material stability and charge transport performance.

Two main factors influence the charge transfer performance of OFETs: charge carrier mobility and injection barrier. The driving force behind electron transfer is the intermolecular interaction between π-π stacked layers, making it desirable to design and synthesize TTF derivatives with extended π-conjugation and high planarity [15]. One way to evaluate the strength of π-conjugation between molecules is to analyze the FMOs, including the highest occupied molecular orbital (HOMO) and the lowest unoccupied molecular orbital (LUMO). It is generally believed that carrier transport occurs between HOMO-n and LUMO+n, and the smaller the energy gap between them, the easier the transport. Additionally, for p-type semiconductors, the energy difference between the work function of the source and drain electrodes and the HOMO level of the OSCs is usually considered the charge injection barrier [16]. A smaller barrier allows for easier charge injection [17], so the HOMO energy level should align with the electrodes’ work function. Clearly, the FMOs of the material play a crucial role in determining the charge transfer performance of OFETs. Consequently, molecular simulation has become an essential tool, helping verify whether the material meets requirements and guiding the design and synthesis of organic semiconductor materials effectively [18].

Recently, theoretical studies have focused on the structural analysis and performance characterization of TTF derivatives, and various simulation methods with different levels of precision have been reported. However, there has been no systematic research on the theoretical calculation methods of the fundamental properties of TTF derivatives. To address this, several typical density functionals were systematically tested to study TTF derivatives, and a compromise between accuracy and computational time was identified. Additionally, a series of TTF derivatives based on benzothiadiazole fused with tetrathiafulvalene was designed. Based on FMOs and reorganization energy, these derivatives are considered strong candidates for sensing OFETs with potential ultra-high charge transport properties.

## 2. Methodology

Over the past few decades, the density functional theory (DFT) method has rapidly advanced and become the primary approach in published studies [19,20]. However, there is no evaluation criteria system among the various DFs for researching TTF derivatives. Therefore, this work examines several typical DFs, including three hybrid-GGA DFs (B3LYP [21], B3PW91 [22], and PBE0 [23]) and one hybrid-meta-GGA DF (M06-2X [24]). Correspondingly, four basis sets are considered: 6-31G(d) [25], def-TZVP [26], 6-311G(d,p), and 6-311+G(d,p) [27]. The hybrid-GGA DFs poorly describe dispersion effects (long-range Coulomb correlation effects) [28]. To better account for intramolecular dispersion, DFT-D3(BJ) [29] dispersion correction is added to the def2TZVP basis set of each DF to address basis set superposition error (BSSE) [25]. The Minnesota functional (M06-2X) is only compatible with D3 in the zero-damping form, as it exhibits mid-range correlations [24]. Additionally, M06-2X performs well in describing weak interactions. Therefore, D3 correction is incorporated into the four basis sets in the M06-2X functional to assess basis set sensitivity.

**Figure 1:**
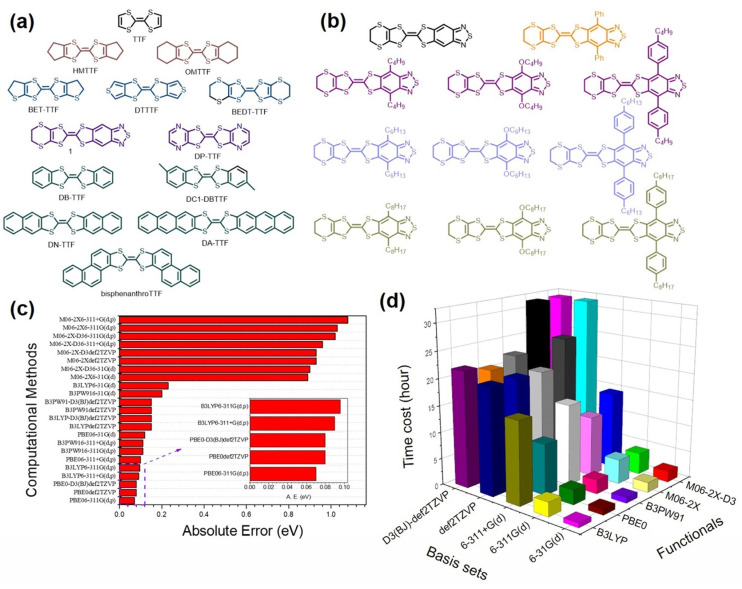
Chemical structural formulas of (**a**) reported typical TTF derivatives and (**b**) designed new TTF derivatives. (**c**) Average absolute errors of theoretical and experimental HOMO energies for the reported TTF derivative molecules of 23 different density functional theory (DFT) methods, used to perform front-line molecular orbital simulation calculations. (**d**) The average time investment of TTF derivative simulation calculations with different DFT methods and different basis sets, where the Z axis is the average time, the front X axis is different basis sets, and the right Y axis is different DFT methods.

In this work, we studied the reported typical TTF derivatives (shown in Figure 1a) and designed new TTF derivatives in Figure 1b. We calculated the average absolute errors of the theoretical and experimental HOMO energies for the studied molecules. For 23 different density functional theory (DFT) methods, we perform front-line molecular orbital simulations on the reported TTF derivatives and compare the theoretical and experimental values to evaluate the accuracy of each method. The experimental values are obtained from the reported literature (measured by cyclic voltammetry). The detailed deviation of HOMO energy values between theoretical calculation and experimental results is listed in Table S1. PBE0/6-311G(d,p) demonstrates the best accuracy in prediction (Figure 1c). Additionally, we analyze the average computational time for TTF derivative simulations using different DFT methods and basis sets (Figure 1d). The detailed CPU consumption time for each simulation calculation method is provided in Table S2. The results are compared with experimental data by balancing accuracy and computational cost. We selected PBE0/6-311G(d,p) [30,31] as our computational method due to its excellent balance of description quality and efficiency for the studied materials. All calculations were performed using Gaussian 09 (Rev. D.01, Gaussian, Inc., Wallingford, CT, 2013), and molecular structures and properties are analyzed with Visual Molecular Dynamics (VMD) software (version 1.9.3) and Multiwfn (version 3.8) [32,33]. The experimental data were obtained from published literature and databases [34,35,36,37].

## 3. Results and Discussion

### 3.1. The Frontier Molecular Orbitals (FMOs)

Based on the PBE0/6-311G(d,p) method, we explore the influence of different substituents on the FMOs of TTF cores and side chains, respectively. It is generally believed that when a molecule reacts with an electrophilic group, the HOMO orbital serves as the reactive site. Similarly, when it reacts with an electron-donating group, the place with LUMO enriched provides a reactive site [38]. Fragments that contribute more to HOMO (LUMO) preferentially combine with positive (negative) charge regions and form charge-transfer complexes in the charge transfer process of organic semiconductors. Therefore, in the concept of molecular design, it is generally believed that the introduction of electron-donating groups (EDG) leads to higher HOMO and LUMO. Similarly, electron-withdrawing groups (EWG) cause lower frontier orbital energy values [13].

#### 3.1.1. FMOs Modification of TTF Derivatives’ Core

**Figure 2:**
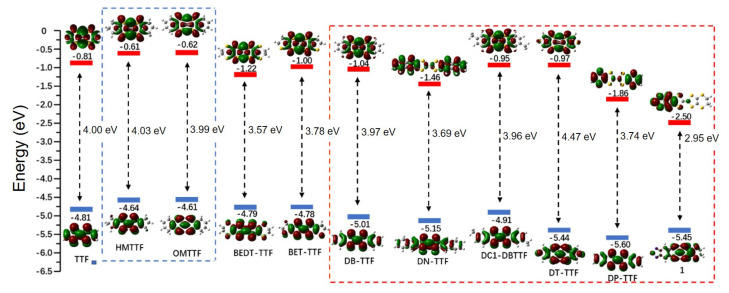
The HOMO/LUMO values and the frontier molecular orbitals of typical TTF derivatives that have been reported. The blue dashed box indicates the introduction of EDG on the TTF core, and the red dashed box indicates the introduction of EWG.

This view can be well illustrated by analyzing the following TTF derivatives by comparing them with the precursor, TTF. For example, the introduction of EDG such as cyclopentane and cyclohexane on both sides of the TTF skeleton can significantly increase the delocalization of electrons (Figure 2), making HMTTF and OMTTF molecules exhibit increased FMOs energy value (HOMO increased from −4.81 eV to −4.64 eV and −4.61 eV, respectively, and LUMO increased from −0.81 eV to −0.61 eV and −0.62 eV, respectively). By introducing EWG on the TTF structure, electron delocalization can be effectively reduced, resulting in a lower FMO energy value. For example, inserting a pyrazine ring into the TTF core is possible because the nitrogen-containing pyrazine ring has a strong electron-withdrawing ability. Compared with TTF, DP-TTF has a lower FMO energy value (HOMO decreases from −4.81 eV to −5.60 eV, and LUMO decreases from −0.81 eV to −1.86 eV). Furthermore, the functionalization of the TTF core with benzene or biphenyl groups, acting as EWG, effectively modulates its frontier orbital energies. The HOMO level is systematically lowered from −4.81 eV in TTF to −5.01 eV in DB-TTF and further to −5.15 eV in DN-TTF, indicating enhanced electronic stabilization. This energy level depression could improve oxidative resistance and their environmental stability in electronic applications. Notably, the introduction of a methyl group to the benzene ring (DC1-DBTTF, HOMO: −4.91 eV) partially counteracts this stabilization through its electron-donating character, demonstrating precise tunability of electronic properties. Incorporating a strongly electronegative S atom into the HMTTF molecule decreases the FMOs energy values of BET-TTF (HOMO drops from −4.64 eV to −4.78 eV, and LUMO drops from −0.61 eV to −1.0 eV). In summary, introducing EDG increases HOMO and LUMO levels, while EWG decreases them; this provides valuable guidance for designing materials with targeted HOMO/LUMO levels.

**Figure 3:**
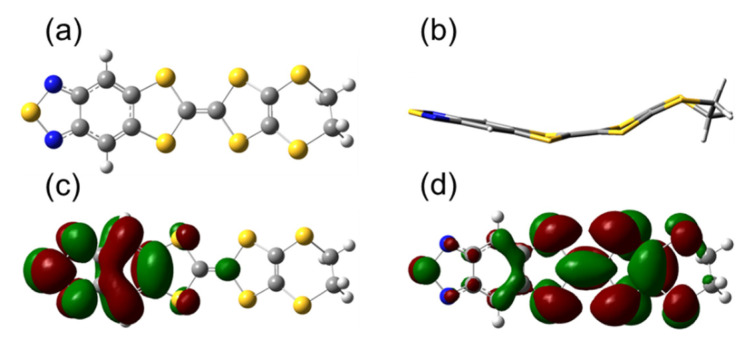
Molecular structure of (**a**) front view and (**b**) side view of molecule 1. The electron cloud distribution maps of (**c**) HOMO and (**d**) LUMO of molecule 1 are shown, obtained from the Gaussian 09 program with PBE0/6-311G(d,p). All calculations are based on PBE0/6-311G(d,p).

To understand the mechanism of how the benzothiadiazole group influences the TTF core, a theoretical computation of the FMOs of molecule 1 was performed as an example. It is known that the strong electron-withdrawing ability of benzothiadiazole can effectively reduce the electron-donating ability of molecules, thereby increasing their air stability [39]. It is clear that introducing benzothiadiazoline causes the HOMO to mainly distribute on the TTF core, while the LUMO is primarily located on the benzothiadiazole (Figure 3). Additionally, from Figure 3b, it can be seen that the molecule has an almost planar structure except for the two -CH_2_ groups at the ends, which greatly facilitates π-π interactions and charge transport. The HOMO/LUMO values and ∆[EHOMO-ELUMO] are −5.45 eV/−2.50 eV and 2.95 eV, respectively, which are much smaller than the −4.81 eV/−0.81 eV and 4 eV values of TTF [40]. A smaller bandgap generally benefits better mobility in organic semiconductor materials [41]. In 2012, Zhu’s research group reported that a thin-film OTFT made from this molecular material achieved a mobility of up to 0.73 cm^2^V^−1^s^−1^ [39], indicating that molecule 1 and its derivatives are excellent candidates for organic semiconductors.

**Figure 4:**
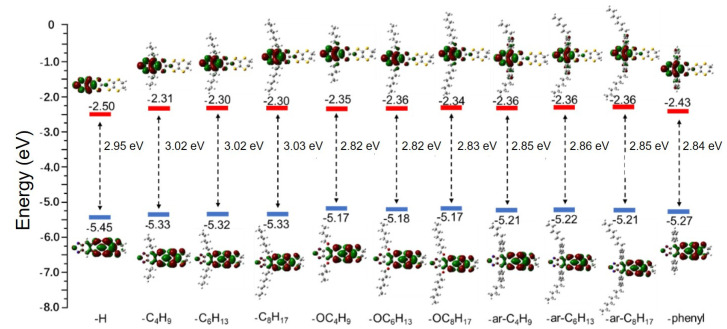
The frontier molecular orbitals maps. The electron cloud distribution map of the FMOs of the newly designed molecule shows blue and red colors, respectively, representing the HOMO and LUMO energy levels. The substituents -H, -C_n_H_2n+1_, -OC_n_H_2n+1_, -ar-C_n_H_2n+1_, and -phenyl, respectively, represent the corresponding substituent groups. All calculations are performed in Gaussian 09 using PBE0/6-311G(d,p).

However, benzothiadiazole fused with tetrathiafulvalene still has numerous shortcomings. Our experiments revealed that it exhibits limited solubility and air stability, which hinder the material’s potential for further applications in sensing and other fields. To address the drawbacks of TTFs, we designed a series of derivatives based on benzothiadiazole fused with tetrathiafulvalene as the molecular backbone (Figure 1b). By substituting various groups at the para position of the six-membered benzothiadiazole ring, we aimed to achieve the desired HOMO/LUMO values for organic semiconductor materials. We introduced four main substituents: straight alkyl chains, benzene rings, oxyalkanes, and straight alkyl chains with benzene rings, as shown in Figure 1b. The structures of all these derivatives were optimized without imaginary frequencies, and their FMO energies were calculated. Firstly, the effect of symmetrical substitution on the derivatives’ structural properties was investigated. We then studied how different substituents influence the structural features of the derivatives (Figure 4). It was found that single-side substituents have lower frontier orbital energies than bilateral ones, especially for benzene ring substituents. One possible explanation is that, compared with strong EWGs, benzothiadiazole acts as an EWG, while the introduced side chain functions as an EDG, which raises the FMO energy levels. In the case of bilateral substitution, the HOMO and LUMO values are generally higher than those of unilateral substitution. The energy gap between them is usually slightly smaller than that of unilateral cases, offering better sensitivity when used in sensor devices. Symmetrical substitution structures were chosen in molecule design because they tend to have higher mobility and improved semiconductor properties [11].

#### 3.1.2. Modification of FMOs with EDG/EWG Side Chains on TTF Derivatives

Here, we first studied the differences in energy levels and electronic distribution. According to Figure 4, we observed that the larger conjugated frame shows a delocalized molecular orbital (MO), such as in a substitution involving a benzene ring. We noted that the same type of substituent groups has little effect on the energy value of the FMOs. Compared with the strong electron-withdrawing ability of benzothiadiazole, three different substituents (-C_n_H_2n+1_, -OC_n_H_2n+1_, -ar-C_n_H_2n+1_) were used as electron-donating groups (EDGs) to increase the electron density of the system, resulting in higher FMO energy values. Especially when the oxyalkanes are used as substituents, the effect is most significant because the electron-donating ability of the conjugation effect of the ether bond surpasses the electron-withdrawing ability of its inductive effect, showing the highest HOMO orbital value (−5.18 eV) and the smallest HOMO-LUMO gap (2.82 eV). The work function close to gold (−5.10 eV) indicates a smaller injection barrier and higher charge transfer, which may guide the design of the targeted molecules.

In addition, we studied the effect of different alkane chain lengths of the same type of substituents on the FMOs. Increasing the length of the carbon chain has little impact on the energy levels of the FMOs. For instance, in the case of bilateral substitution, whether the straight-chain alkane with lengths of four, six, or eight carbon atoms, the HOMO and LUMO levels remain nearly the same. However, generally, increasing the side chain alkane length appropriately can improve the material’s solubility [13,42], which is beneficial for large-scale, low-cost production methods like solution processing [43]. Additionally, the length of the alkane chain may influence the material’s stability and sensitivity [44], which requires further verification in upcoming experiments. Therefore, for this type of derivatives, we can tailor the target molecule by adjusting the alkane chain length based on specific needs. Adding different substituents impacts the energy levels of the FMOs to varying degrees. Compared to benzothiadiazole fused with tetrathiafulvalene, this molecular design aims to enhance solubility, stability, and charge transfer in experiments. All these factors indicate that these derivatives are promising candidates for organic semiconductor materials.

### 3.2. Reorganization Energy

It is generally believed that charge jumps between adjacent molecules to facilitate the charge transfer (CT) process from the Marcus charge transfer mechanism [45]. The CT rate constant (W) can be described by the Marcus–Hush Equation (1):

(1) $$W\;=\;{\left(\frac{\pi }{\lambda k_{B}T}\right)}^{\frac{1}{2}}\exp(-\frac{\lambda }{4k_{B}T})\;$$

Among them, λ is the reorganization energy, kB is the Boltzmann constant, V is the effective charge transfer coupling between adjacent molecules in the single organic crystal, T is the temperature, and H is the Planck constant. The equation shows that the charge transfer rate is closely related to the reorganization energy λ and the charge transfer integral V [46]. It is generally believed that small reorganization energy and large charge coupling correspond to considerable carrier mobility. The reorganization energy measures the energy change of the system caused by the relaxation of the geometric structure after the electronic state changes. We predict the possible charge transfer mechanism of the newly designed molecules and how the substituents affect charge transfer performance. The reorganization energy calculation procedure is illustrated in Figure S1.

**Figure 5:**
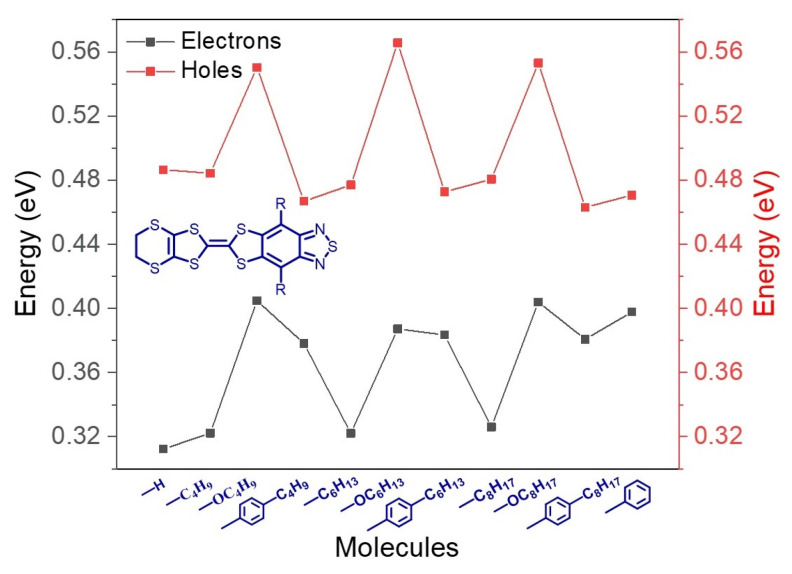
The impact of various substituent groups on the hole and electron reorganization energy of the molecules, where R indicates the position of the substituent, and the horizontal axis shows the different substituents.

The calculation results are shown in Figure 5. The influence of the substituent groups on the reorganization energy of holes and electrons displays the same trend. We found that when the side-chain substituent group R is a straight alkane (-C_n_H_2n+1_), the corresponding reorganization energy is the smallest. Conversely, when the substituent group is oxyalkanes (-OC_n_H_2n+1_), the reorganization energy is the largest. This can be explained by the steric hindrance effect of the structure [47]. For simple alkane chain substitution, when the carrier jumps, the molecular core undergoes noticeable structural relaxation. However, because of the significant steric hindrance between the straight alkane chain and the benzene ring of the parent nucleus, the side substituent groups only exhibit simple in-plane stretching with minimal swings.

The situation changes completely when the oxygen (O) atom is introduced to form a substituent with an ether bond. Besides allowing more structural relaxation of the core part away from the plane, the steric hindrance between the side substituent groups and the core is significantly decreased due to the O atom’s presence. During carrier injection, we observe that the -C_4_H_9_ substituent group swings along with the oxygen atom, leading to greater structural relaxation and reorganization energy. At the same time, we find that the hole reorganization energy of this newly designed molecule is higher than the corresponding electron reorganization energy, indicating a better electron transport channel than holes. The overall structural relaxation from the neutral to the ionized state is greater than from the neutral to the anionic state, suggesting that this group of materials could be used in n-type devices [48]. While these theoretical calculations are predicated on idealized conditions. However, in practical applications, the performance of OFET devices depends not only on the selection of the organic semiconductor material but also on various additional factors. These factors encompass the morphology of the thin film, its crystallinity, interfacial properties, and the fabrication techniques, such as vapor deposition or solution processing [49].

### 3.3. Study of Binding Energy in Gas Detection

**Scheme 1:**
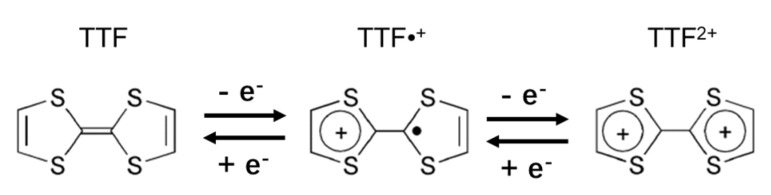
Reversible oxidation state from TTF to TTF•+ and TTF^2^+.

A key application of OFETs is gas monitoring. Related organic materials are used in OFETs to detect SO_2_, NO_2_, and other gases [48,50]. When organic materials interact with gas molecules, these molecules can influence the electronic properties through mechanisms such as forming traps, acting as dopants, or creating resistive interface barriers. This interaction alters the intermolecular forces between molecules in the organic semiconductor (OSC) and the dielectric [51], which affects the device’s output parameters. The TTF and its derivatives are reported as electroactive and strong π-donors, with their redox potentials sensitive to the gas molecules that exist in the environment. They act as electron donors and undergo electron transfer by capturing the gas molecules in the environment, which can be transformed into TTF•^+^ or even TTF^2+^, thereby changing the source-drain current of the semiconductor layer of the OFET devices (shown as Scheme 1). The electron-accepting or electron-donating groups in the molecule can facilitate the capture of reducing or oxidizing gases, achieving high sensitivity [52]. For example, Yang and coworkers reported benzothiadiazole-fused tetrathiafulvalene (BEDT-TTF)-based OFET devices and their capability to detect toxic nerve agents like diethyl chlorophosphite and POCl_3_ vapors, with a detection limit of 10 ppb [39]. Researchers have also developed sensors that incorporate TTF and its derivatives into frameworks. For example, Jiang and colleagues report an NH_3_ gas sensor made from porous zirconium metal−organic frameworks (Zr-MOFs) that serve as inorganic nodes, with redox-reversible TTF acting as organic linkers [53].

**Figure 6:**
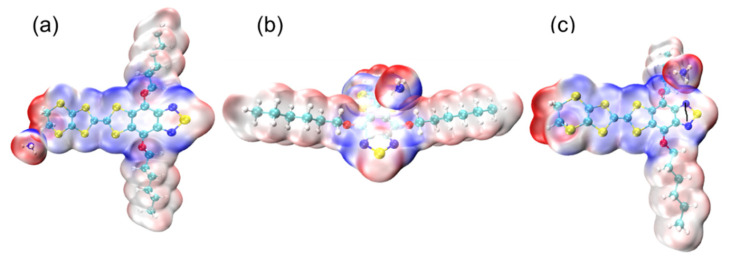
Electrostatic potential adsorption configurations of the TTF derivative and ammonia molecules. (**a**) The lowest-energy structure, showing N–C non-covalent bonding. (**b**,**c**) Less stable configurations, with energies 0.07 eV and 0.03 eV higher than (**a**), respectively.

Electrostatic potential (ESP) on the van der Waals surface is a useful tool for identifying reaction sites, molecular properties, and hydrogen bonding interactions [54]. As an example, we consider the adsorption of NH_3_ by an organic molecule with the substituent -OC_6_H_13_ in a newly designed material. First, we use the Multiwfn program [33] to calculate and visualize the electrostatic potential diagram of the organic and gas molecules. Based on the principle of mutual attraction between positive and negative potentials, several possible conformations were initially proposed, as shown in Figure 6.

Figure 6 displays electrostatic potential adsorption diagrams of TTF derivative and ammonia molecules at different relative positions. The adsorption model is selected based on the lowest conformational energy, with the lowest energy system being 0.07 eV and 0.03 eV lower than configurations b and c, respectively. In this configuration, the negative surface potential of the nitrogen (N) atom in NH_3_ and the positive surface potential of C-H bonds in organic molecules interact, indicating the formation of N-C non-covalent bonds. According to the analysis of the Reduced Density Gradient (RDG) [55], the N atom in the ammonia molecule and the hydrogen (H) atom at the end of the organic molecule form a weak hydrogen bond.

**Figure 7:**
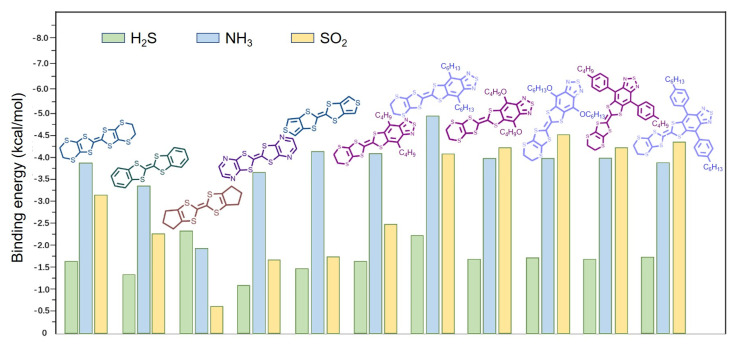
Comparison of binding energy calculations between small organic molecules and gas molecules. The five on the left are common TTF derivative molecules that have been reported, and the last six are newly designed molecules in this work. The colors indicate different gas molecules: green, blue, and yellow represent binding energy calculations with H_2_S, NH_3_, and SO_2_, respectively.

We calculated the adsorption binding energies of the typical TTF derivatives and newly designed molecules according to this principle. These gases include H_2_S, NH_3_, and SO_2_. The smaller the value, the more stable the corresponding composite conformation and the stronger the binding ability [56]. The specific calculation results are shown in Figure 7.

Compared to the typical TTF derivatives that have been reported, it is clear that the newly designed molecules exhibit stronger adsorption and sensitivity to these gas molecules. This is especially true for SO_2_. It should be noted that we only performed simulations using the dimer model. In real gas-filled environments, differences in gas adsorption are more pronounced. The binding energy calculations also confirm that the newly designed TTF derivative materials are excellent candidates for OSC materials. Calculating binding free energy offers valuable guidance for designing functional OFET devices.

## 4. Conclusions

In this work, the theoretical methods used to simulate the TTF derivatives system are systematically studied, and PBE0/6-311G(d,p) has been identified as the most cost-effective option. First, the influence of different substituent groups on the frontier orbital energy is analyzed, including EDGs (such as alkane chains, double bonds, triple bonds, etc.) that enhance the FMO energy by increasing electron delocalization, and EWGs (such as nitrogen rings and benzene rings) that reduce electron delocalization and thus lower the FMO energy. Second, we provide theoretical guidance for designing molecules with ideal HOMO/LUMO levels and present the side chains of benzothiadiazole fused with tetrathiafulvalene as a new set of TTF derivatives. Moreover, these TTF derivatives are predicted to have narrower HOMO-LUMO energy gaps than traditional benzothiadiazole fused with tetrathiafulvalene, with HOMO values that closely match the work function of metal electrodes, indicating smaller injection barriers and improved performance. Finally, the lower electron reorganization energy suggests that these materials could be potential N-type semiconductor materials. The comparison of binding energies for three types of gases suggests they could serve as good sensor-OTFT candidates for targeted SO_2_ detection.

## Data Availability

Data will be made available on request.

## Supplementary Materials

The following supporting information can be downloaded at: https://www.mdpi.com/article/10.3390/s25196190/s1, Table S1. Deviation of HOMO energy values between theoretical calculation and experimental results (eV). Table S2. CPU consumption time of each simulation calculation method (h). Figure S1. The calculation steps of the hole reorganization energy, the reorganization energy is expressed as λ = λ(I) + λ(II), where λ(I) = |E2 − E1|, λ(II) = |E3 − E4|, where E1 is the energy of the neutral state under the structure of the neutral state, E2 is the energy of the cation state under the structure of the neutral state, E3 is the energy of the neutral state under the structure of the cation state, and E4 is the energy of the cation state under the structure of the cation state. Electronic reorganization can follow the same calculation steps, except that the charged state becomes −1.
