# Supplementary material for: Molecular Design of Benzothiadiazole-Fused Tetrathiafulvalene Derivatives for OFET Gas Sensors: A Computational Study
Source: Sensors (Basel). 2025 Oct 6;25(19):6190. doi: 10.3390/s25196190 (PMC12526700; doi:10.3390/s25196190)
Supplement: Supplementary file 1 [file sensors-25-06190-s001.zip › sensors-3849644-supplementary.pdf]

Supporting information

Molecular Design of Benzothiadiazole-Fused Tetrathiafulvalene Derivatives for OFET Gas Sensors: A Computational Study

Xiuru Xu * and Changfa Huang

Key Laboratory of Optoelectronic Devices and Systems of Ministry of Education and Guangdong Province, College of Physics and Optoelectronic Engineering, Shenzhen University, Shenzhen, 518000, China
* Correspondence: xiuruxu@szu.edu.cn

Table S1. Deviation of HOMO energy values between theoretical calculation and experimental results (eV)

Functional	Basis sets	TTF	HMTTF	DT-TTF	DB-TTF	DN-TTF	BEDT-TTF	DP-TTF	BET-TTF	OMTTF	average error
B3LYP	6-31G(d)	0.22	0.33	0.06	0.3	0.29	0.54	0.11	0.35	0.34	0.28
B3LYP	6-311G(d,p)	0.03	0.14	0.11	0.11	0.1	0.35	-0.06	0.16	0.15	0.13
B3LYP	6-311+G(d,p)	-0.03	0.09	-0.17	0.05	0.04	0.32	-0.14	0.13	0.1	0.12
B3LYP	def-TZVP	0.12	0.21	0.02	0.18	0.16	0.47	-0.02	0.28	0.24	0.19
B3LYP-D3(BJ)	def-TZVP	0.13	0.21	0.02	0.19	0.17	0.48	-0.01	0.29	0.24	0.19
M06-2X	6-31G(d)	-0.93	-0.81	-1.06	-0.86	-0.86	-0.66	-1.02	-0.83	-0.83	0.87
M06-2X	6-311G(d,p)	-1.07	-0.99	-1.22	-0.99	-0.99	-0.78	-1.13	-0.96	-0.96	1.01
M06-2X	6-311+G(d,p)	-1.12	-1.03	-1.27	-1.04	-1.05	-0.8	-1.19	-0.98	-0.99	1.05
M06-2X	def-TZVP	-0.98	-0.85	-1.05	-0.92	-0.92	-0.67	-1.08	-0.84	-0.87	0.9
M06-2X-D3	6-31G(d)	-0.93	-0.87	-1.06	-0.86	-0.86	-0.66	-1.02	-0.83	-0.84	0.88
M06-2X-D3	6-311G(d,p)	-1.07	-0.94	-1.22	-0.99	-0.99	-0.78	-1.13	-0.96	-0.96	1.0
M06-2X-D3	6-311+G(d,p)	-1.12	-0.97	-1.28	-1.04	-1.05	-0.8	-1.19	-0.98	-0.99	1.04
M06-2X-D3	def-TZVP	-0.98	-0.85	-1.05	-0.92	-0.92	-0.67	-1.08	-0.84	-0.87	0.9
PBE0	6-31G(d)	0.07	0.15	-0.14	0.13	0.11	0.37	-0.07	0.18	0.17	0.15
PBE0	6-311G(d,p)	-0.07	0.01	-0.26	-0.01	-0.03	0.24	-0.18	0.05	0.03	0.09
PBE0	6-311+G(d,p)	-0.13	-0.06	-0.31	-0.06	-0.08	0.22	-0.25	0.02	-0.01	0.12
PBE0	def-TZVP	0.02	0.06	-0.12	0.06	0.03	0.36	-0.13	0.17	0.11	0.11
PBE0-D3(BJ)	def-TZVP	0.02	0.08	-0.12	0.07	0.04	0.37	-0.13	0.18	0.11	0.12
B3PW91	6-31G(d)	0.2	0.28	0	0.25	0.23	0.51	0.05	0.32	0.3	0.23
B3PW91	6-311G(d,p)	0.06	0.13	-0.12	0.11	0.09	0.38	-0.07	0.19	0.16	0.14

B3PW91	6-311+G(d,p)	0	0.09	-0.17	0.06	0.04	0.36	-0.13	0.16	0.12	0.12
B3PW91	def-TZVP	0.14	0.21	0.01	0.18	0.15	0.49	-0.02	0.3	0.24	0.19
B3PW91-D3(BJ)	def-TZVP	0.15	0.20	0	0.19	0.16	0.5	-0.01	0.3	0.24	0.19

Table S2. CPU consumption time of each simulation calculation method (h)

Functional	Basis sets	TTF	HMTTF	DT-TTF	DB-TTF	DN-TTF	DP-TTF	BET-TTF	OMTTF	Bisphenanthro TTF	Average time
B3LYP	6-31G(d)	0.01	0.01	0.2	0.25	0.61	0.2	0.33	2.4	3.61	0.84
B3LYP	6-311G(d,p)	0.01	5.6	0.36	0.53	1.4	0.4	0.65	6.73	8.1	2.64
B3LYP	6-311+G(d,p)	0.2	12.9	0.86	1.43	4.51	0.88	1.65	17.2	99.6	15.47
B3LYP	def-TZVP	0.01	38.2	2.31	3.48	10.6	2.48	4.63	40.0	79.3	20.1
B3LYP-D3(BJ)	def-TZVP	0.01	40.4	2.3	3.85	9.5	2.9	4.65	53.5	79.7	21.8
M06-2X	6-31G(d)	0.11	4.1	0.3	0.41	0.93	0.35	0.48	4.53	5.2	1.82
M06-2X	6-311G(d,p)	0.01	15.3	0.48	0.8	2.0	0.58	1.11	9.45	11.4	4.57
M06-2X	6-311+G(d,p)	0.01	17.4	1.0	1.6	4.55	1.26	2.5	26.5	46.7	11.28
M06-2X	def-TZVP	0.78	57.9	3.21	4.5	10.6	3.23	6.1	57.8	83.7	25.3
M06-2X-D3	6-31G(d)	0.11	3.4	0.33	0.43	0.93	0.35	0.48	7.68	5.1	2.09
M06-2X-D3	6-311G(d,p)	0.16	10.0	0.48	0.8	2.28	0.58	1.1	8.83	11.7	3.99
M06-2X-D3	6-311+G(d,p)	0.31	24.5	1.0	1.53	4.61	1.25	2.5	22.93	71.1	14.4
M06-2X-D3	def-TZVP	0.78	76.6	3.0	4.25	10.7	3.21	6.1	96.8	83	31.6
PBE0	6-31G(d)	0.06	1.2	0.2	0.25	0.63	0.2	0.33	3.0	3.6	1.05
PBE0	6-311G(d,p)	0.13	4.7	0.36	0.48	1.45	0.4	0.68	8.5	8.0	2.74
PBE0	6-311+G(d,p)	0.21	15.2	0.9	1.45	5.1	0.8	1.68	18.2	42.4	9.54
PBE0	def-TZVP	0.63	35.7	2.56	3.65	9.38	2.85	4.73	44.0	79	20.2
PBE0-D3(BJ)	def-TZVP	0.65	40.1	2.58	3.56	8.55	2.86	4.75	44.0	79.5	20.7
B3PW91	6-31G(d)	0.01	1.8	0.2	0.26	0.61	0.2	0.31	2.66	3.58	1.07
B3PW91	6-311G(d,p)	0.01	6	0.28	0.48	1.38	0.4	0.65	6.1	8	2.58
B3PW91	6-311+G(d,p)	0.2	12.2	0.88	1.43	5.0	0.9	1.71	16.0	99.2	15.2
B3PW91	def-TZVP	0.01	39	2.56	3.81	9.48	2.86	4.7	40.2	79.1	20.19
B3PW91-D3(BJ)	def-TZVP	0.01	49.8	2.6	3.61	9.38	2.93	4.75	47.3	80	22.26

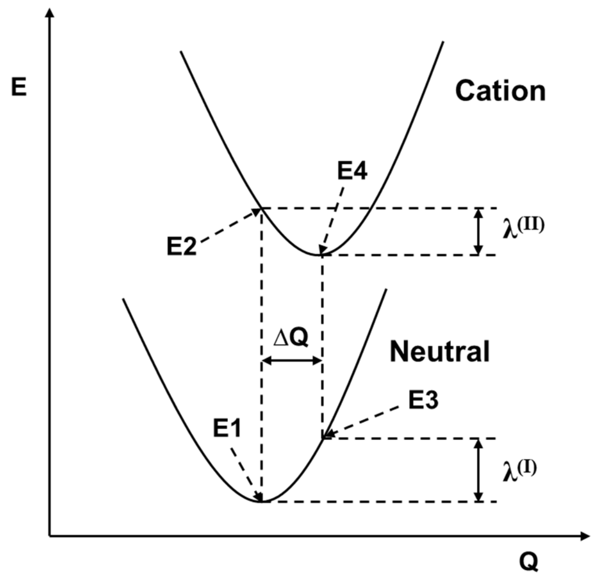

Figure S1. The calculation steps of the hole reorganization energy, the reorganization energy is expressed as $\lambda=\lambda(I)+\lambda(II)$, where $\lambda(I)=|E2-E1|$, $\lambda(II)=|E3-E4|$, where E1 is the energy of the neutral state under the structure of the neutral state, E2 is the energy of the cation state under the structure of the neutral state, E3 is the energy of the neutral state under the structure of the cation state, and E4 is the energy of the cation state under the structure of the cation state. Electronic reorganization can follow the same calculation steps, except that the charged state becomes -1.
